# The experience of teaching introductory programming skills to bioscientists in Brazil

**DOI:** 10.1371/journal.pcbi.1009534

**Published:** 2021-11-11

**Authors:** Luíza Zuvanov, Ana Letycia Basso Garcia, Fernando Henrique Correr, Rodolfo Bizarria, Ailton Pereira da Costa Filho, Alisson Hayasi da Costa, Andréa T. Thomaz, Ana Lucia Mendes Pinheiro, Diego Mauricio Riaño-Pachón, Flavia Vischi Winck, Franciele Grego Esteves, Gabriel Rodrigues Alves Margarido, Giovanna Maria Stanfoca Casagrande, Henrique Cordeiro Frajacomo, Leonardo Martins, Mariana Feitosa Cavalheiro, Nathalia Graf Grachet, Raniere Gaia Costa da Silva, Ricardo Cerri, Rommel Thiago Juca Ramos, Simone Daniela Sartorio de Medeiros, Thayana Vieira Tavares, Renato Augusto Corrêa dos Santos

**Affiliations:** 1 São Carlos Institute of Physics, University of São Paulo, São Carlos, Brazil; 2 Department of Genetics, Luiz de Queiroz College of Agriculture, University of São Paulo, Piracicaba, Brazil; 3 Department of General and Applied Biology, São Paulo State University, Rio Claro, Brazil; 4 Center of the Study of Social Insects, Department of General and Applied Biology, Institute of Biosciences of Rio Claro, São Paulo State University, Rio Claro, Brazil; 5 Ribeirão Preto Medical School, University of São Paulo, Ribeirão Preto, Brazil; 6 Department of Computer Science, Federal University of São Carlos, São Carlos, Brazil; 7 School of Natural Sciences, Universidad del Rosario, Bogotá, Colombia; 8 Computational, Evolutionary and Systems Biology Lab, Center for Nuclear Energy in Agriculture, University of São Paulo, Piracicaba, Brazil; 9 Regulatory Systems Biology Lab, Center for Nuclear Energy in Agriculture, University of São Paulo, Piracicaba, Brazil; 10 Barretos Cancer Hospital, Barretos, Brazil; 11 Paulista School of Medicine, Federal University of São Paulo, São Paulo, Brazil; 12 Department of Genetics, Evolution, Microbiology and Immunology, Institute of Biology, University of Campinas, Campinas, Brazil; 13 Genomics for Climate Change Research Center, University of Campinas, Campinas, Brazil; 14 Roche Sequencing Solutions, Pleasanton, California, United States of America; 15 Department of Infectious Diseases and Public Health, Jockey Club College of Veterinary Medicine and Life Sciences, City University of Hong Kong, Hong Kong, Special Administrative Region, People’s Republic of China; 16 Institute of Biological Sciences, Federal University of Pará, Belém, Brazil; 17 Department of Informatics and Statistics, Federal University of Santa Catarina, Florianópolis, Brazil; 18 Department of Genetics and Evolution, Federal University of São Carlos, São Carlos, Brazil; 19 School of Pharmaceutical Sciences of Ribeirao Preto, University of São Paulo, Ribeirão Preto, Brazil; 20 Institute of Biology, State University of Campinas, Campinas, Brazil; SIB Swiss Institute of Bioinformatics, SWITZERLAND

## Abstract

Computational biology has gained traction as an independent scientific discipline over the last years in South America. However, there is still a growing need for bioscientists, from different backgrounds, with different levels, to acquire programming skills, which could reduce the time from data to insights and bridge communication between life scientists and computer scientists. Python is a programming language extensively used in bioinformatics and data science, which is particularly suitable for beginners. Here, we describe the conception, organization, and implementation of the Brazilian Python Workshop for Biological Data. This workshop has been organized by graduate and undergraduate students and supported, mostly in administrative matters, by experienced faculty members since 2017. The workshop was conceived for teaching bioscientists, mainly students in Brazil, on how to program in a biological context. The goal of this article was to share our experience with the 2020 edition of the workshop in its virtual format due to the Coronavirus Disease 2019 (COVID-19) pandemic and to compare and contrast this year’s experience with the previous in-person editions. We described a hands-on and live coding workshop model for teaching introductory Python programming. We also highlighted the adaptations made from in-person to online format in 2020, the participants’ assessment of learning progression, and general workshop management. Lastly, we provided a summary and reflections from our personal experiences from the workshops of the last 4 years. Our takeaways included the benefits of the learning from learners’ feedback (LLF) that allowed us to improve the workshop in real time, in the short, and likely in the long term. We concluded that the Brazilian Python Workshop for Biological Data is a highly effective workshop model for teaching a programming language that allows bioscientists to go beyond an initial exploration of programming skills for data analysis in the medium to long term.

## Author summary

Bioscientists analyzing research data deal with challenges because most lack computer science background, such as programming skills, making it difficult to process their data and communicate with data analysts. Over the last few years (2017 to 2020), we assembled interdisciplinary teams of graduate and undergraduate students to develop the Brazilian Python Workshop for Biological Data. These short courses aimed to teach programming skills in a real-world setting. They were offered in Portuguese to facilitate accessibility to both Brazilians and foreigners doing research in Brazil. We accomplished these goals by emphasizing both basic programming skills and foundational concepts, alongside hands-on activities designed with biological datasets. Importantly, we were supported by experienced faculty. Although the first editions were in-person, we reformulated the 2020 edition to an online version due to the Coronavirus Disease 2019 (COVID-19) pandemic. During the online 2020 edition, we taught using a variety of tools to facilitate synchronous and asynchronous communication between participants and organization and to engage participants in activities that promoted their active participation and networking. We used digital notebooks and encouraged students to put into practice shareable and reproducible research. In 2020, we also performed online surveys with participants that helped us to implement real-time improvements and perspectives of future changes based on the students’ feedback. Our workshop comprises a model for future initiatives.

## Introduction

Today, bioscientists are dealing with an unprecedented amount of data, which requires knowledge of computer science basic competencies. There is an increasing demand for training life scientists, especially in Latin American countries such as Brazil, which are slowly developing in bioinformatics research compared to other nations. In Brazil, training in this area has been promoted in different formats, from completely dedicated graduate programs to semester-long and short hands-on courses. These initiatives have been held by institutions, associations, and teams of trainers. In 2017, we conceived the Brazilian Python Workshop for Biological Data as a means to ameliorate the lack of training opportunities for undergraduate and graduate students in Brazil (details about our motivation are presented on **S1 Text**). This initiative organized by students has been encouraged and supported by professors in computational biology and data science. The workshop has been taught in Portuguese, the native language of most organizers and students, and was designed for life science researchers of different backgrounds with no prior, or with limited knowledge, in any programming language. Instead of teaching specific analysis pipelines, our course introduced basic computer science concepts using the Python language to analyze real-world biological datasets. We chose Python as it has a low barrier to entry because of its human-readable syntax, it is applicable to automate a wide range of data analyses, and it has become increasingly popular among established bioinformaticians, as with some of our trainers. A detailed description of the overall aspects and organization over the years is provided in **S2 Text**.

Over the years, the workshop improved empirically and from “transferable skills” of organizers. In the first workshop (2017), the organizing team comprised graduate and undergraduate students that had prior experience in analysis of biological data (e.g., by engaging in research projects in life sciences). They were aware of the need for bioscientists and wanted to hand on some basic knowledge to bioscientists without programming skills. Even though these organizers had little prior practical experience in running training events, some contributed previously to The Carpentries workshops (https://carpentries.org/) and were supported by experienced professors. Experience was also gained empirically by instructors participating in the organization over the years, in either teaching programming skills and in the content suitable for being taught in only a few days of workshop. In addition, complementary to this empirical learning, since 2020, a research environment consisting of reading and discussing literature that describe similar initiatives (e.g., teaching programming to bioscientists) has been encouraged among interested instructors, which allowed increased critical mass in teaching programming to bioscientists. This approach contributed to writing this manuscript (coauthors in this manuscript include members of the organizing team of the 2017, 2018, and 2020 editions) and to discussions on how our initiative could be improved over time. The workshop benefited the community as it brings teaching and communication skills to organizers and is likely to improve “computational thinking” and awareness of the importance of reproducibility in science among participants (more details on the main takeaways are presented on **S3 Text**).

**Fig 1:**
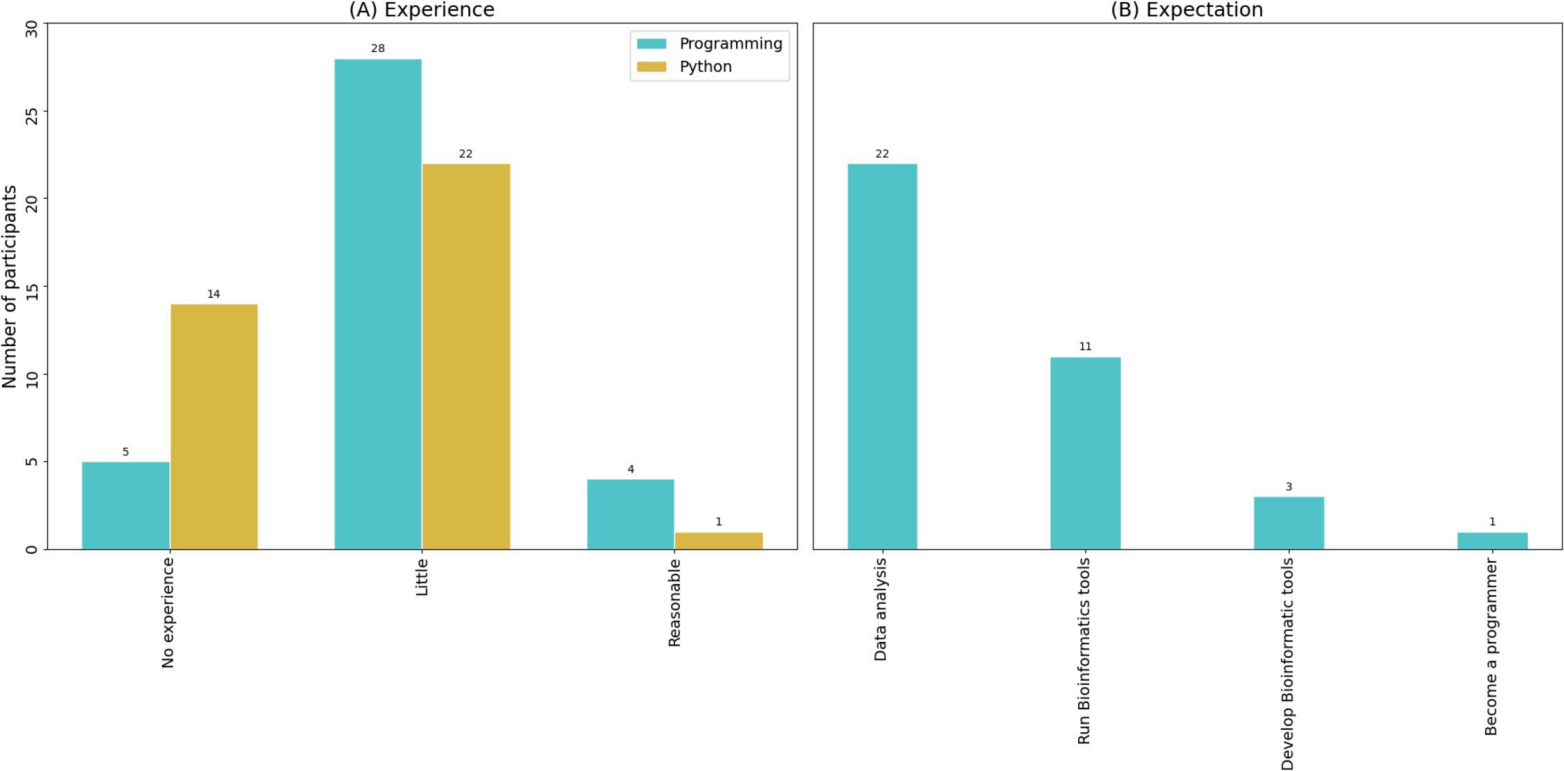
**(A)** Previous programming knowledge of participants. Most participants reported little or no experience with either programming in general (blue) or python in particular (yellow). **(B)** Attendees’ expectations concerning the type of knowledge to be gained during Brazilian Python Workshop for Biological Data in 2020.

In this manuscript, we describe the online 2020 edition of the Brazilian Python Workshop for Biological Data. The organizing team was composed of 19 people, including graduate and undergraduate students, and professors. A total of 37 students attended the workshop. We selected these learners based on preestablished criteria: involvement in scientific research in which computational analysis of biological data is expected, with little or no prior programming skills (**Fig 1A**); with expectations to analyze biological data, but not to run or develop bioinformatics tools, or become programmers (experts) (**Fig 1B**). The criteria we adopted are of particular relevance, because we could select students with a solid background in biology and with the expectation to learn basic programming skills, which is aligned with our purposes (avoiding frustrations from both students and organizers). It is also important to mention that we invite students from previous editions with potential to use Python in their projects in life sciences to participate as organizers in future workshops, as a means to maintain this initiative alive and active.

**Fig 2:**
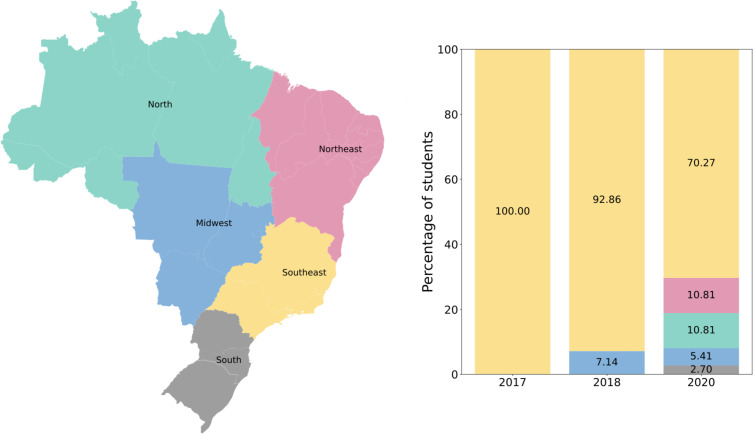
Brazil map to show the geographic distribution of participants who attended the Brazilian Python Workshop for Biological Data over the years (https://www.ibge.gov.br/geociencias/organizacao-do-territorio/malhas-territoriais/15774-malhas.html?=&t=acesso-ao-produto).

Differently from previous editions, the 2020 workshop had to be redesigned to an online format due to the social distance restrictions imposed by the Coronavirus Disease 2019 (COVID-19) pandemic. In this context, here, we present the strategies we adopted for converting the workshop from an in-person to a virtual event and the advantages of doing so. For instance, industry was less likely to sponsor, but our costs significantly reduced (e.g., no coffee breaks, lunch, or lodging costs). A more inclusive environment was possible for the members of the organizing team, speakers, and participants. Most participants were graduate and undergraduate students enrolled in biological sciences courses (e.g., genetics, molecular biology, or biomedicine) from institutions in different regions in Brazil (**Fig 2**).

The following sections of the manuscript cover topics on course structure, content, teaching approach, and learning metrics. We also report the assessment of the virtual workshop by the participants and how it helped the organizers reflect on short and long-term improvements. We discuss the challenges conquered and why this format may be a reasonable strategy to be adopted.

## Workshop materials and presentations

### Workshop structure of the virtual edition (2020)

As many teaching initiatives recommend [1,2], Python was chosen because it is an open-source, interpreted, general purpose, and multiparadigm programming language [3]. General and specific bioinformatics tools and libraries have been developed in Python. Moreover, those libraries are regularly updated and supported by a large community of experts. These factors contribute to the choice of Python as a language for bioinformatics and for teaching beginners [4].

The workshop in 2020 was structured to cover 4 days of immersive Python learning (**S1 Table**). Each day started with an introductory talk, followed by an interactive lecture in which students were encouraged to ask questions and perform tasks. The afternoons consisted of a seminar to show students advanced applications of programming skills in biological sciences (**S2 Table**) or a networking event (an hour of conversation on day 2 to establish a friendly atmosphere as we believe this would improve the learning experience), followed by a practical session including group exercises. We reserved the last hour of each day for questions and clarifications of concepts introduced during classes. A final group challenge and a flash talk happened in the afternoon of the fourth day. In this activity, 3 participants explained their ongoing research projects in a 20-minute presentation, followed by a group discussion about how their data could be analyzed using Python. This session helped promote further engagement in biological data analysis.

**Fig 3:**
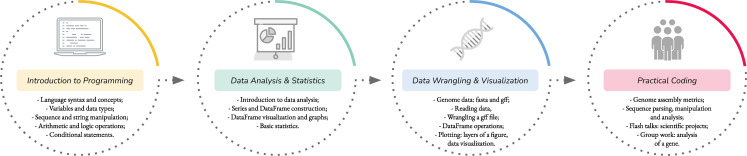
Overview of the workshop topics and activities involving learned skills over the 4 days of the Brazilian Python Workshop for Biological Data in 2020 (detailed schedule of the workshop is provided as S1 Table and all the Python built-in functionalities and libraries used are provided in S3 Table).

On the first day of the event, participants were introduced to variables, data types, arithmetic, logic operations, and conditional statements. Lists and strings were used to provide participants the notion of sequential data. An example-based approach was used during the rest of the event to ensure that participants were able to understand the usage of the Python programming language in a biological context (**Fig 3**). We adopted this methodology during the 3 editions of the workshop with slight adjustments.

We used Pandas [5] and Matplotlib [6] during the workshop due to their wide usage across the data science community. We also used Biopython [7] for its software community development and because it provides a wide range of tools specifically designed for biological data analysis. The live coding approach was employed to give participants an overview of the range of methods and functions and practical usages of these libraries. After the first introduction of a given library, we set goals for students to employ the libraries they have learned. In fact, we used Pandas and Matplotlib during the second day as tools to help in performing statistical analyses (**Fig 3, Box 1**). Given the importance of wrangling in biological data, topics related to these libraries were reinforced during the remaining days of the event. On day 3, Pandas’ usage was consolidated by wrangling a genome annotation file to obtain information on exons in a tabular format (**Fig 3**). On the same day, Matplotlib was used to reinforce concepts of figure, subplot, axes, and axis. By understanding the different layers behind Matplotlib graphs, participants were able to create custom graphs. Finally, the fourth day had a practical section for evaluating a genome assembly using common Python modules and specific Biopython modules for dealing with biological sequences. During all practical sessions, we encouraged participants to share ideas and suggestions on how to achieve the results of each exercise or task.

**Box 1. Topics covered on each day of the workshop. More information, including details on the schedule and libraries/functions used during the 4 days of the event, is available in S1 and S3 Tables**

First day

The first day was a 5-hour introduction to basic programming concepts and data structures. Students were encouraged to organize their notebooks using text chunks and also comments in their codes. After introducing the programming language, we defined variables, data types, and conversion, followed by the arithmetic and logical operations. These topics paved the way for conditional statements, where students could learn how to build the structures of comparisons and code execution. We then covered sequence manipulation, as well as understanding the usage of built-in methods and functions. The last topics involved output formatting and handling.

- Variables
- Data types and conversion
- Arithmetic operations
- Logical operations
- Conditional statements
- Sequence manipulation
- Built-in methods and functions
- Output formatting and handling

Second day

We focused on data manipulation and data visualization for statistical analyses. To this end, we taught students how to import tabular data in both series and data frames containing attributes to be analyzed. We performed data conversion, string handling, and selection and also showed how to combine information from different tables by joining and merging methods. Techniques for aggregation and group operations were used mainly for descriptive statistics, while other mathematical functions were used for calculation and obtaining confidence intervals. We also included a topic for data export in spreadsheets for saving the final results. For a graphical summary, we explored examples of figures and discussed which plots are more suitable according to the type of result. We spent 5 hours covering these topics, although we reviewed some of those concepts during the following days.

- List comprehension
- Data import and export
- Series/DataFrame construction and attributes
- Data conversion
- String handling
- Data wrangling
- Descriptive statistics
- Data visualization
- Mathematical functions

Third day

The third day comprised a 5-hour in-depth analysis of biological data. The focus was wrangling genome annotation data and calculating descriptive statistics about genomic features (e.g., introns). Concepts worked throughout the first days were reviewed—data import and export, series and data frames, string handling, and data conversion and manipulation—and novel topics were addressed. In the data handling section, we made use of the repetition structures for automatizing a common task for a large number of observations. Besides tabular format, results were also compiled into figures, where we presented in detail the structure of plot layers, customization, and combination of plots.

- Repetition structures
- Data import and export
- Series/DataFrame construction and attributes
- Data conversion
- String handling
- Data wrangling
- Descriptive statistics
- Data visualization

Fourth day

The last day of the event had 2 hours of live coding focused on evaluating a genome assembly. We reviewed data import, output formatting and handling, and repetition structures. For sequence manipulation, we included topics for parsing, handling, content analysis, extracting attributes in FASTA files, and counting hashable objects. The last activity comprised 2 hours of hands-on sequence analysis, where groups of students should identify the most suitable commands to solve the task.

- Repetition structures
- Output formatting and handling
- Sequence parsing and handling
- Object attributes
- Sequence content analysis
- Handling genome sequence file
- Data import
- Counting hashable objects

### Using the Google Colab digital notebooks

Communication happened on the Slack platform (an organized texting/media sharing environment designed to facilitate group collaboration; https://slack.com), whereas lectures occurred via Google Meet (a group video conference and screen sharing tool). As the target audience was beginners in programming, we used digital notebooks for coding, which makes it a more accessible and interactive process. Notebooks allow users to organize code development using cells that contain code, text, plots, media, or mathematics [8]. We chose to work with Jupyter Notebooks during the first 2 editions of the workshop mainly due to its wide usage in the data science community [9]. The process involved the configuration of the notebooks using a local installation of Anaconda (an open source management system that installs, runs, and updates packages easily; https://docs.conda.io) in students’ laptops before the event. As the 2020 edition was online, we implemented Google Colab notebooks that run Python code through the browser, do not require installation, and run entirely on the cloud. In this way, the use of Google Colab may democratize the accessibility of the workshop activities.

The use of the Google Colab platform was also useful for material preparation. Google Colab is built upon a Jupyter Notebook with the addition of collaborative features, which make it easy for sharing and adding comments. Furthermore, the notebook is automatically saved in the registered Google account, and it can be accessed remotely (https://colab.research.google.com/). Its intuitive interface also made possible the contribution of members of the organizing committee with no programming skills. It is important to highlight that digital notebooks facilitate the reproducibility of data analysis, which is particularly important for science [9,10].

### Strategies for an online event on biological data analysis

Challenges inherent to transitioning the workshop to an online format required changes in the course structure and inclusion of more interactive activities throughout the event. Participants were encouraged to interact with peers and instructors on Slack, which was an efficient platform for asking questions and for communicating technical issues in real time. Throughout the workshop, it was interesting to notice that some of the participants were able to answer questions from their peers as they became familiarized with coding.

Participants showed interest in applying the acquired knowledge to analyze biological data from publicly accessible databases. We covered data analysis from epidemiology (number of infections and deaths caused by COVID-19 in Brazil) (https://en.wikipedia.org/wiki/COVID-19_pandemic_in_Brazil/Statistics), botany (allelopathic effect of *Casuarina equisetifolia* extraction on seed germination and growth information from 4 crop plants) [11], genetics and genomics (introns and exons data of *Schistosoma mansoni*) [12]. Evolution and other genomic-related topics were presented during seminars, in which examples of research and in-depth applications of Python in life sciences were discussed (**S2 Table**).

### Individual and group exercises

To evaluate the participants’ improvement in Python programming skills, they were asked to do individual and group exercises. An example of an individual activity was to document one of the lectures using the Google Colab platform, using comments throughout the notebook. This activity aimed to assure their commitment during lectures and to encourage good reproducibility practices. From a total of 33 notebooks submitted by participants for evaluation, 32 showed good documentation and note-taking in the form of comments and/or markdown text. It showed us that students were able to make connections between learned concepts, carry out specific analyses, and report and discuss the results.

**Fig 4:**
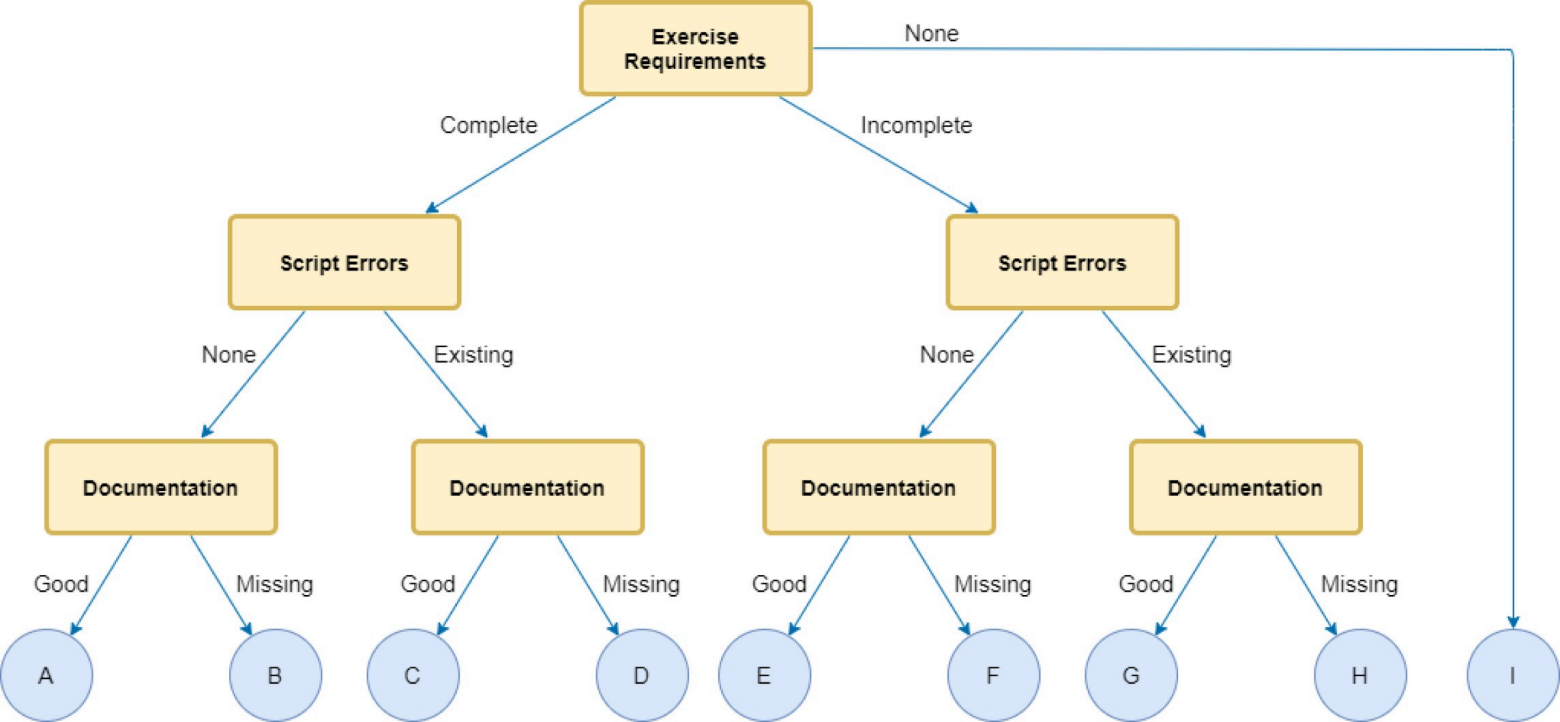
Decision tree demonstrating how rubrics were used to classify notebooks in the final group activity.

The group activity consisted of analyzing the genome locus with the *SlrA* protein-coding gene from the biofilm-producing bacteria *Bacillus subtilis* (NCIB 3610/ATCC 6051). The sequence was available in a Google Drive (a cloud-based storage solution) folder as a text file. Participants were expected to import this sequence file into a Google Colab Notebook and to analyze it using the libraries presented during the workshop, 3 or more Python basic data structures, and at least 1 of Python control structures and/or comparison operators. Group leaders, who were a participant in the group most comfortable with this role, were encouraged to present the group code and explain logic behind it. Notebooks were later categorized according to the completion of exercise requirements, script errors, and documentation (**Fig 4**). Five groups were classified in category A (complete notebooks, without script errors, and good documentation), 1 in B category (complete exercise and no script errors, but no documentation), and 1 in F category (no script errors, but missing documentation and incomplete exercises). Only 1 group was unable to complete the exercise due to issues while uploading the required data (category I). Moreover, 6 groups (85.7%) completed all the exercise requirements, 5 (71.4%) made good code documentation, and all 7 (100%) wrote scripts with no errors.

## Assessment of quality and impact of the workshop

Although several organizers have been gaining experience over the years from delivering programming skills and from informal interaction with participants in the first events (2017 and 2018), the workshop in 2020 was the first edition in which we collected formal feedback from participants. Our 2 main objectives were (i) to collect daily feedback from participants, which allowed us to promptly deliver a better experience in real time and reflect on the experience in the long term; and (ii) to provide us with knowledge of the general training experience, quality, and impact of the workshop. In total, all 37 participants agreed to respond to all forms. It is important to note that not all questions were answered by every student because not all questions were mandatory.

We benefited greatly from the learning from learners’ feedback (LLF), i.e., regular collection of feedback that allowed workshop adaptability (feedback forms in **S4 Table**). During the workshop, organizers took action to overcome adverse conditions based on these feedbacks. For instance, one of the instructors had internet connectivity issues during the practical session, so the next day, we provided the lesson material that was missed. We used the LLF to respond in real time to the participants’ needs. We could quickly assess whether explanation of concepts, live coding, and instructor’s pace were being delivered satisfactorily (**Table 1**).

**Table 1:** Quotes from students with feedback obtained from daily and final surveys (quotes have been translated from Portuguese).

Question	Answer
Daily feedback	
How was the experience of group work? In your opinion, what is the importance of this type of interaction for learning?	“Very good. It is highly important because some problems or errors are visualized and resolved more easily with the work group.” “It was a good experience. Also, to asking questions and sharing experience, we seek for answers together, learning from each other.” “I believe that the activity carried out today did not make a significant difference.”
How has been the experience of using platforms of communication during the Workshop (Slack and Google Meet)? In your opinion, did this type of interaction is useful in your learning?	“It helped a lot, even to interact more with colleagues. The only complication is to change screens all the time, this is a little distracting [. . .] The fact that questions have to be asked on Slack makes us lose the thread on Google meetings.” “It’s been good. Yes, the Slack channels made it possible for archive the information, to access later.” “Yes. Slack makes communication a lot easier, not to mention that leaves each subject separated in channels. [. . .] In addition, it allows you to send different kind of files, such as images. This make it easier to show the code error [. . .].”
How do you evaluate the experience of writing your own codes? Do you believe it helped in your learning?	“Undoubtedly, writing the code itself is essential for learning. When we make a mistake, we already know what to look at next time as a possible error. Also, writing my own codes makes the learning process more practical and improves our logics.” “I found it difficult, mainly because I had to copy it live and in a short time, but I believe with practice it will flow better.”
What is your opinion about the flash talk session? Do you think it was relevant to contextualize the use of Python in biological data analysis?	“Extremely important. Bringing practice to our reality is essential to understand the magnitude of programming in the biological field.” “I think it was not so interesting considering the theme of the Workshop. The presentations were more related to the themes of each project and not to the use of Python in them.”
Final evaluation	
How much or how the contents presented during the event are applied to your research?	“Everything, from data organization to the final result will be used.” “[. . .] will be used to manipulate DNA sequence for genomics and evolution [. . .].” “[. . .] to the raw data of sequencing, and graphics construction.”
What was the experience of participating in a distance learning course? Would you take another course in the same format?	“The experience was great. I think the course was very well adapted to the distance version. I would definitely do another one in this format.” “I would. I think it did not affect learning and help with the financial issue (travel and accommodation).” “I would, but I confess that you lose a little bit of concentration.”

Another important gain from the LLF was assessing how appropriate the workshop was for the selected participants. Their feedback allowed us to understand if their expectations were aligned with the scope of the workshop. In addition, their feedback revealed organizational shortcomings, which will be used to improve future initiatives to deliver content more effectively.

The LLF approach greatly benefitted the organizers in our successful transition to a virtual event. However, it was an exhaustive process for participants. In live coding sessions, for instance, participants had to have 2 browsers open, one for Google Meet, and another for their Google Colab Notebooks. They also had to monitor Slack from time to time and frequently report their feedback, which required another browser window. Understandably, it was bothersome and distracting (**Table 1**). In order to mitigate this problem, we allocated time in the agenda specifically for reporting feedback.

We provided a final evaluation form (questions in **S5 Table**) to assess the overall quality of the new online format of the workshop. It was challenging to keep participants engaged, actively participating, and away from distractions [13]. We split the participants into groups for daily exercises and to work together on a group challenge on the last day. We aimed to include people with different experience levels mainly by balancing the number of graduate and undergraduate students. With very few exceptions, participants reported that these activities were constructive and that troubleshooting errors was helpful. Regarding the flash talk experience, participants’ feedback revealed that this activity helped contextualize how Python can be applied in scientific research. Moreover, students emphasized that it enriched the range of applications they could envision (**Table 1**).

Another important problem reported was conflicts in academic schedules, which required the participants to plan ahead their enrollment in the workshop. We believe that schedule was an important reason why students did not enroll in the workshop.

We were able to evaluate how participants enjoyed their experience, the creation of their own code, and the hands-on coding sessions. Unlike the survey analyzed on ELIXIR training experiences [14], we regret that our proposal in 2020 did not include an evaluation of the long-term workshop consequences on participants’ lives. However, the final evaluation provided valuable material for organizers, who could reflect on the initiative over the years. The student’s feedback showed that they were delighted with the live coding sessions. Many of them emphasized their ability to code grew during these sessions mainly because the instructors taught them extensively how to troubleshoot errors.

At the end of the workshop, we asked participants to self-assess their improvement, for which the results were very optimistic. In the final assessment, the number of participants with good programming skills grew by almost 65%. Several participants recognized there was an alignment between their research analysis and the content they learned in the workshop. Regarding their experience in an online workshop, most students reported the workshop was very well adapted to the virtual mode as 87.9% of participants reported being open to attending another online course, with 18.2% stating that they just participated because it was online.

Besides the positive feedback, we look forward to enhancing participant engagement. We were delighted to hear that the instructions were clear, the pace was adequate, and that our instructors were enthusiastic and very helpful. However, one important concern was the workload, as only 58.8% reported “excellent.” Our solutions to tackle this problem include (i) to reduce the overall content of the workshop; (ii) to spread the workshop over more days; and (iii) to allocate more time for hands-on activities. In future workshop events, we will plan a more adequate workload for the content being delivered. Additionally, we must strategize a better management of software employed so that their virtual experience in the workshop is not jeopardized. Another suggestion was to use the same dataset for all the hands-on activities. However, a variety of datasets increases their exposure to the data and might be useful for other bioscientists. Finally, students also requested a session on data management and organization, which is in alignment with our aims to include more content that benefits the science’s quest for reproducibility. Although we emphasized the importance of carefully documenting the data analysis, and several initiatives in Brazil already exist envisioning better data management (e.g., the São Paulo Research Foundation: https://fapesp.br/gestaodedados), our future plans will be to include the culture and practice of best data management toward reproducibility.

The Brazilian Python Workshop for Biological Data comprises a suitable model for teaching a programming language and encourages bioscientists to go beyond an initial exploration of data analysis in the medium to long term. Based on our 5-year experience and future perspectives, we provide a set of recommendations for those planning similar workshops (**Box 2**). We were able to successfully transition to an online format without compromising the quality that our event cultivated over the years. The LLF approach employed was extremely valuable and allowed us to excel during this unprecedented year.

**Box 2. Distilled set of recommendations for those planning to run similar workshops**

**Activities implemented in the online workshop**

- Stay online if the format benefits the community. Due to COVID-19 impositions, we had to adopt a virtual event, but the online format allowed us to reach a more diverse group of participants because the enrollment from different regions in Brazil increased.
- Know your audience. Be specific regarding the scope of the workshop early on. Select participants based on predefined criteria that fit the scope, which will avoid participant frustrations and course dropout.
- Establish a friendly atmosphere. Allocate time in the agenda for networking opportunities, such as online lunch breaks. We also encourage everyone to introduce themselves and interact via Slack throughout the workshop.
- Keep data science democratic and reproducible. Implement the use of notebooks that are great tools for sharing code. These notebooks, such as Jupyter and Google Colab, have an intuitive interface and allow code commenting and markdown. In our case, we used Google Colab that have all these features and the benefit that it is completely web based, not requiring local installation.
- Ensure students’ commitment during lectures. Encourage participants to ask questions and to share ideas. We plan cooperative learning activities such as group exercises and a final group challenge.
- Bet on interactive teaching approaches. Employ example-based learning methods throughout the sessions, particularly in the hands-on live coding portion.
- Encourage good code documentation by setting standards. Ask participants to keep good code documentation by making use of comments and markdown cells explaining each step throughout the notebook.
- Remove the language barrier. Opt to conduct the workshop in the native language of your audience when possible. In our case, we use Portuguese, with the objective to facilitate the learning process for students that are not comfortable with English (the default language in programming).
- Be available to help. Have organizers that are free to help students when they need, in addition to instructors. Choose a platform like Slack where you can create group channels and private chats to accommodate participant’s personalities, e.g., extroverts and introverts, and that will enhance their interactions, particularly when asking questions. Try to promptly answer questions and to allocate time in the agenda for general review and Q&A sessions at the end of each day.
- Learn from your learners’ feedback. Obtain daily feedback from participants to guide the decisions to improve the event in real time.
- Keep the community growing. Select participants from previous editions to be organizers, which helps to maintain the initiative alive and active.
- Have a diverse and strongly compromised organizing team. Organizers are not just instructors. Several organizers are behind the scenes providing support on several topics, such as media coverage, ethical committee, and helping with course material.
- Bring experts with real-world experience. Include invited speakers in the agenda. They can be professors and scientists who currently apply the tools we teach in the workshop in their daily research.

**Our plans for future editions (virtual or in-person)**

- Make a blind selection of participants. Scientific social networks are very small! Apply filters in the selection aligned with the expectations of the workshop scope, but do not provide the names and other information that can bias your decision.
- Be inclusive. Ask for information like gender, ethnicity, geographic location, etc., in the enrollment form and use such information during selection to increase diversity.
- Be more accessible. Provide written transcripts, alternative text for visual content, and adapt color blind pallets. In addition, enquiring about a participant’s disability or accessibility needs to be translated into improvements for the student’s experience, such as providing a sign language translator.
- Take time to breathe. Plan short breaks during sessions and longer breaks between sessions, which also allows time for participants to fill up feedback forms. Avoid repetitively doing the same type of activities or exercise during a session.
- Have clear directions for exercises and activities. Firstly, determine what is the object of the activity, and set out what exactly the participants need to accomplish with the activity. For instance, (i) plan an activity with the objective of practicing reproducible code; (ii) participants need to demonstrate they added comments in the code and markdown cells to the notebook. Lastly, make sure to provide a clear and detailed explanation of the activity during the session followed by the expectations of the organizers, especially if grading is involved.
- Teach about reproducibility in data analysis. Invite speakers to talk about well-established protocols in good data and programming practices and project management that enable reproducible data science. Add hands-on sessions of good data practices and reproducibility.
- Reduce the workload for organizers. Automate the evaluation of exercises in which we expect a specific response from multiple lines of code. This is particularly suitable in workshops with a large number of participants or with a low instructor to participants ratio. An example of a Python library that can be used for this purpose is *nbgrader* (https://nbgrader.readthedocs.io).

## Ethics statement

The study was approved by the Human Research Ethics Committee of the ESALQ (Protocol number 5395—USP—Escola Superior de Agricultura "Luiz de Queiroz”) and was registered at the Brazilian Ethical Office (Plataforma Brasil: 33159820.6.0000.5395). All participants provided consent through an online form prior to the beginning of the workshop, and their confidentiality was ensured during data collection by replacing names with alphanumeric codes.

## Supporting information

S1 Table Schedule of the Brazilian Python Workshop for Biological Data in 2020. (DOC)

S2 Table Seminars that introduced students to advanced applications of programming skills in biological science. (DOC)

S3 Table Python concepts and libraries introduced to students in the 2020 edition. (DOC)

S4 Table Questions addressed daily to students who attended the 3rd edition of the workshop (2020). (DOC)

S5 Table Questions addressed in the final evaluation survey to students who attended the 3rd edition of the workshop (2020). (DOC)

S6 Table General information and statistics from the Brazilian Python Workshops for Biological Data over the years. (DOC)

S1 Text Need for bioinformatics training in Brazil and Latin America. (DOC)

S2 Text A summary of the Brazilian Python Workshops for Biological Data. (DOC)

S3 Text Final remarks about the virtual workshop during the COVID-19 pandemic and main takeaways. COVID-19, Coronavirus Disease 2019. (DOC)
