# Supplementary material for: The experience of teaching introductory programming skills to bioscientists in Brazil
Source: PLoS Comput Biol. 2021 Nov 11;17(11):e1009534. doi: 10.1371/journal.pcbi.1009534 (PMC8584955; doi:10.1371/journal.pcbi.1009534)
Supplement: S1 Table — (DOC) [file pcbi.1009534.s001.doc]

**S1 Table. Schedule of the Brazilian Python Workshop for Biological Data in 2020.**

| **Time** | **Day 1** | **Day 2** | **Day 3** | **Day 4** |
| --- | --- | --- | --- | --- |
| 8h30 | Welcome | Presentation: Statistics in Python | Presentation: Problem of the Day | Presentation: Problem of the Day |
| 9h00 | Live Coding - Introduction to Python and its data structures | Live Coding - Introduction to Pandas and Statistics | Live Coding - Wrangling a Genome Annotation Data | Live Coding - Evaluation of a Genome Assembly |
| 10h00 |  |  |  |  |
| 11h00 |  |  |  |  |
| 12h00 | Break | | | |
| 13h00 | Talk - Applications of Python to Biological Data: The Example of the PloidyNGS Tool | Networking Event | Talk - Python for Evolution Studies: From Simulation to Data Analysis | Talk - Metrics for Evaluating a Genome Assembly With Python |
| 14h00 | Live Coding - Introduction to Python and its data structures | Live Coding and Exercises - Introduction to Pandas and Statistics | Live Coding and Exercises - Wrangling a Genome Annotation Data | Flash Talks - Participants Projects |
| 15h00 |  |  |  | Final Group Activity - Sequence Analysis Using Python |
| 16h00 |  |  |  |  |
| 17h00 - 18h00 | Questions and Answers | | | |

# 
