# Supplementary material for: The experience of teaching introductory programming skills to bioscientists in Brazil
Source: PLoS Comput Biol. 2021 Nov 11;17(11):e1009534. doi: 10.1371/journal.pcbi.1009534 (PMC8584955; doi:10.1371/journal.pcbi.1009534)
Supplement: S2 Table — (DOC) [file pcbi.1009534.s002.doc]

**S2 Table. Seminars that introduced students to advanced applications of programming skills in biological science.**

| **Edition** | **Talks** | |
| --- | --- | --- |
|  | **Keynote speakers** | **Title** |
| **2017** | Prof. Dr. Flávia Vischi Winck | Biological problems and computational solutions |
|  | Prof. Dr. Diego Mauricio Riaño  Pachón | (Some) biological problems and computational approaches |
| **2018** | Dr. Anally Ribeiro da Silva Menegasso | Applications of Proteomic Analysis |
|  | - | Corporate scientist. How to navigate in this world? |
|  | Prof. Dr. Renato Vicentini dos  Santos | Systems Biology and Genetic Regulatory Networks |
|  | Dr. Marcelo Falsarella  Carazzolle | Applications of Bioinformatics in Biotechnology |
|  | - | Automatization in Biotech |
|  | Prof. Dr. Ricardo Cerri | A Framework using Machine Learning to identify and classify transposable elements |
|  | - | Using Python for linear regression from Molecular Markers data (SNPs) |
| **2020** | Prof. Dr. Diego Mauricio Riaño  Pachón | Python application in biological data: the example of the "PloidyNGS" tool |
|  | Dr. Andréa T. Thomaz | Python in evolutionary studies: from simulations to data analysis |
|  | Prof. Dr. Rommel Ramos | Evaluation metrics of genome assembly with Python |
