# Supplementary material for: The experience of teaching introductory programming skills to bioscientists in Brazil
Source: PLoS Comput Biol. 2021 Nov 11;17(11):e1009534. doi: 10.1371/journal.pcbi.1009534 (PMC8584955; doi:10.1371/journal.pcbi.1009534)
Supplement: S3 Table — (DOC) [file pcbi.1009534.s003.doc]

**S3 Table. Python concepts and libraries introduced to students in the 2020 edition.**

| **Tool** | **Topics** | **Day 1** | **Day 2** | **Day 3** | **Day 4** |
| --- | --- | --- | --- | --- | --- |
| Python built-in functionalities | Variables | X |  |  |  |
|  | Data Types and Conversion | X |  |  |  |
|  | Arithmetic Operations | X |  |  |  |
|  | Logical Operations | X |  |  |  |
|  | Conditional Statements | X |  |  |  |
|  | Sequence Manipulation | X |  |  |  |
|  | Build-In Methods and Functions | X |  |  |  |
|  | Repetition Structures |  |  | X | X |
|  | Output Formatting and Handling | X |  |  | X |
|  | List Comprehension |  | X |  |  |
| Pandas | Data Import and Export |  | X | X |  |
|  | Series/DataFrame Construction and Attributes |  | X | X |  |
|  | Data Conversion |  | X | X |  |
|  | String Handling |  | X | X |  |
|  | Data Selection |  | X | X |  |
|  | Data Joining and Merging |  | X | X |  |
|  | Data Aggregation and Group Operations |  | X | X |  |
|  | Descriptive Statistics |  | X | X |  |
|  | Plotting |  | X |  |  |
| Matplotlib | Figure Layers and Structure |  |  | X |  |
|  | Figure Customization |  |  | X |  |
|  | Sub Plot |  |  | X |  |
|  | Bar Plot |  | X |  | X |
|  | Histogram Plot |  |  | X |  |
|  | Line Plot |  | X | X |  |
|  | Box Plot |  |  | X |  |
|  | Saving Figures |  |  | X |  |
| Biopython | Sequence Parsing |  |  |  | X |
|  | Sequence Handling |  |  |  | X |
|  | Object Attributes |  |  |  | X |
|  | Sequence Content Analysis |  |  |  | X |
|  | Extracting Fasta and Multi-Fasta Data |  |  |  | X |
| Numpy | Mathematical Functions |  | X |  |  |
| Scipy | Probability Density Function and Confidence Interval |  | X |  |  |
| Requests | Data Import |  |  |  | X |
| Collections | Counting Hashable Objects |  |  |  | X |
