# Supplementary material for: The experience of teaching introductory programming skills to bioscientists in Brazil
Source: PLoS Comput Biol. 2021 Nov 11;17(11):e1009534. doi: 10.1371/journal.pcbi.1009534 (PMC8584955; doi:10.1371/journal.pcbi.1009534)
Supplement: S4 Table — (DOC) [file pcbi.1009534.s004.doc]

**S4 Table. Questions addressed daily to students who attended the 3rd edition of the workshop (2020).**

| **Question** | **Day** | **Answer** |
| --- | --- | --- |
| How does today’s lecture increase your perception of programming applications in bioscience? | 1 and 3 | Open field for long response |
| In summary, how could the class's knowledge be applied to your research project? | 2, 3 and 4 | Open field for long response |
| How was the group work experience? In your opinion, what is the importance of this kind of interaction for learning? | 2 | Open field for long response |
| In your opinion, was there any problem with group activity? If so, please let us know. | 2 | Open field for long response |
| How has the experience of using communication platforms been during the Workshop (Slack, Google Meet chat)? In your opinion, did this kind of interaction improve your learning? | 3 | Open field for long response |
| How do you evaluate the experience of writing your own codes? Do you believe it helped in your learning? | 4 | Open field for long response |
| What is your opinion about the Flash Talks session? Do you think it was relevant to contextualize the use of Python in the analysis of biological data? | 4 | Open field for long response |
| In your opinion, was any subject more difficult? If so, please let us know. | All | Open field for long response |
| There was any topic that should be reviewed? Which one? | All | Open field for long response |
| What are the criticisms and suggestions of the day? | All | Open field for long response |
