# Supplementary material for: The experience of teaching introductory programming skills to bioscientists in Brazil
Source: PLoS Comput Biol. 2021 Nov 11;17(11):e1009534. doi: 10.1371/journal.pcbi.1009534 (PMC8584955; doi:10.1371/journal.pcbi.1009534)
Supplement: S5 Table — (DOC) [file pcbi.1009534.s005.doc]

**S5 Table. Questions addressed in the final evaluation survey to students who attended the 3rd edition of the workshop (2020).**

| **Question** | **Answer** |
| --- | --- |
| How do you evaluate your level of dedication to the course? | Option: Excellent, Good, Regular, Poor |
| How do you evaluate your level of knowledge at the BEGINNING of the course? | Option: Excellent, Good, Regular, Poor |
| How do you evaluate your level of knowledge at the END of the course? | Option: Excellent, Good, Regular, Poor |
| Do you think the course contributed to improve your skills/knowledge? | Option: Excellent, Good, Regular, Poor |
| Were the instructors efficient? | Option: Excellent, Good, Regular, Poor |
| Were the presentations clear and organized? | Option: Excellent, Good, Regular, Poor |
| Did the instructors stimulate students' interest? | Option: Excellent, Good, Regular, Poor |
| Did the instructors use their time well during classes? | Option: Excellent, Good, Regular, Poor |
| Were the instructors accessible and helpful? | Option: Excellent, Good, Regular, Poor |
| Were the objectives clear? | Option: Excellent, Good, Regular, Poor |
| Was the course content organized and well planned? | Option: Excellent, Good, Regular, Poor |
| Was the course load appropriate? | Option: Excellent, Good, Regular, Poor |
| Was the course organized to allow the participation of all students? | Option: Excellent, Good, Regular, Poor |
| Was the teaching material well prepared? | Option: Excellent, Good, Regular, Poor |
| Were the tools adequate for the course? | Option: Excellent, Good, Regular, Poor |
| Regarding attendance in the workshop: did any commitment to graduate or postgraduate courses, or technical problems, prevent your participation on any days? If so and if it is possible to share, which one? | Open field for long response |
| Which aspects of this course were most useful? | Open field for long response |
| How much the contents presented during the event applied to your research? | Open field for long response |
| How would you improve this course? | Open field for long response |
| Do you have any other comments, criticisms or suggestions? | Open field for long response |
| Is there anything you would like to include in the course? | Open field for long response |
| How was the experience of attending distance learning course? Would you take another course in the same format? | Open field for long response |
| (Optional) Do you have any additional notes about the event? Would you like to write a personal report? Please use this space. | Open field for long response |
