# Supplementary material for: The experience of teaching introductory programming skills to bioscientists in Brazil
Source: PLoS Comput Biol. 2021 Nov 11;17(11):e1009534. doi: 10.1371/journal.pcbi.1009534 (PMC8584955; doi:10.1371/journal.pcbi.1009534)
Supplement: S6 Table — (DOC) [file pcbi.1009534.s006.doc]

**S6 Table. General Information and statistics from the Brazilian Python Workshops for Biological Data over the years.**

|  | **2017** | **2018** | **2020** |
| --- | --- | --- | --- |
| **Number of Applicants** | 90 | 174 | 350 |
| **Number of Enrolled Students** | 20 | 29 | 37 |
| **Number of sponsors involved** | 6 | 4 | No sponsors involved |
| **Support** | Chemistry Institute - University of São Paulo (IQ/USP)  Laboratory of Regulatory Systems Biology, University of São Paulo | International Society for Computational Biology Regional Student Group Brazil  (RSG-Brazil)  Foundation for Institutional Support for Scientific and Technological Development of the Federal University of São Carlos (FAI-UFSCar) | Luiz de Queiroz Agrarian Studies Foundation (FEALQ)  Scientific Communication group of the Department of Genetics (GENt)  Genetics and Plant Breeding Group "Prof. Roland Vencovsky" (GVENCK) |
| **University/Departments involved** | Chemistry Institute - University of São Paulo (IQ/USP) | Computer science department - University of São Carlos (UFScar) | Nuclear Energy Center of University of São Paulo (CENA/USP) and Agriculture College “Luiz de Queiroz” of University of São Paulo (ESALQ/USP) |
| **Number of persons involved in organization** | 10 | 14 | 19 |
| **Number of days** | 2 days | 4 days | 4 days |
