# Supplementary material for: The experience of teaching introductory programming skills to bioscientists in Brazil
Source: PLoS Comput Biol. 2021 Nov 11;17(11):e1009534. doi: 10.1371/journal.pcbi.1009534 (PMC8584955; doi:10.1371/journal.pcbi.1009534)
Supplement: S1 Text — (DOC) [file pcbi.1009534.s007.doc]

Supplementary material 1

**Luíza Zuvanov^1,¶^**, **Ana Letycia Basso Garcia^2,¶^**, **Fernando Henrique Correr^2,¶^**, **Rodolfo Bizarria Júnior^3,9,¶^**, Ailton Pereira da Costa Filho**^4^**, Alisson Hayasi da Costa**^5^**, Andréa T. Thomaz**^6^**, Ana Lucia Mendes Pinheiro**^2^**, Diego Mauricio Riaño-Pachón**^7^**, Flavia Vischi Winck**^8^**, Franciele Grego Esteves^9^, Gabriel Rodrigues Alves Margarido**^2^**, Giovanna Maria Stanfoca Casagrande^10^, Henrique Cordeiro Frajacomo**^5^**, Leonardo Martins^11^, Mariana Feitosa Cavalheiro^12,13^, Nathalia Graf Grachet^14^, Raniere Gaia Costa da Silva^15^, Ricardo Cerri**^5^**, Rommel Thiago Juca Ramos^16^, Simone Daniela Sartorio de Medeiros^17^, Thayana Vieira Tavares^18^, **Renato Augusto Corrêa dos Santos^*,19,20^**

* [renatoacsantos@gmail.com](mailto:renatoacsantos@gmail.com)

**^¶^** these authors contributed equally

[Demand for bioinformatics training in Brazil and Latin America](#_heading=h.1fob9te)

[References](#_heading=h.3znysh7)

# Demand for bioinformatics training in Brazil and Latin America

Bioinformatics emerged back in the 1950s, before DNA sequencing became feasible. At that time, the use of computational techniques for analyzing biological data was largely inaccessible due to difficulties in accessing computers. Nowadays, bioinformatics has become a growing topic due to affordability of computers with user-friendly interfaces, the existence of high-level programming languages and software that facilitate biological data analysis. At the same time, the unprecedented volume of data produced by routine experimental methods and the demand for analyses of complex datasets coupled with holistic interpretation of results, require bioscientists to have a wide range of skills, in which programming has special relevance [[1](https://paperpile.com/c/zTap62/3FeN)].

In order to fulfill the demands raised by researchers to have computer-related competencies, several initiatives were created and have been promoting bioinformatics training and learning. The Global Organisation for Bioinformatics Learning, Education and Training (GOBLET), the Pan African Bioinformatics Network for the Human Heredity and Health in Africa (H3ABioNet) consortium [[3](https://paperpile.com/c/zTap62/Oi4W)], the Carpentries ([www.carpentries.org](http://www.carpentries.org)) and the CABANA (<https://www.cabana.online/index>), comprise few of many examples of such initiatives.While formal curricula at universities are of great importance, *ad hoc* efforts, from a few hours to semester-long research projects, workshops and courses, are being pivotal in the immediate gain of computational skills by scientists [[2](https://paperpile.com/c/zTap62/jLtU)]. In Latin America (LA), the number of publications in bioinformatics is below the world average [[4](https://paperpile.com/c/zTap62/DkFY)]. Perspectives of bioinformatics in several countries of LA previously emphasized their needs and challenges, presenting promising perspectives of growth [[5–7](https://paperpile.com/c/zTap62/7hiD+qvnV+2leC)], but it has still being recognized that the growth of the area in general, and programming and mathematical skill in particular, has been slower than in other parts of the world (<https://www.ebi.ac.uk/about/news/press-releases/bioinformatics-training-latin-america> ). In Brazil, a network of laboratories was created in the 1990s for genome sequencing of important bacterial plant pathogens, which boosted bioinformatics in the country. Additionally, research on important crops, such as sugarcane and eucalyptus, as well as animal and human-health related research, contributed significantly to the advances of bioinformatics in Brazil. In 2006, although several advances in research had been recorded, educational initiatives in Brazil included only the graduate programs in Bioinformatics at the University of São Paulo (USP) and Federal University of Minas Gerais (UFMG), together with the National Laboratory for Scientific Computation (LNCC) [[7](https://paperpile.com/c/zTap62/2leC)].

After more than a decade, there is still a limited number of graduate school programs training in bioinformatics, such as the Bioinformatics Interunits Program of the Federal University of Minas Gerais (<http://www.pgbioinfo.icb.ufmg.br/>) and Graduate Program in Bioinformatics of the University of São Paulo (https://www.ime.usp.br/en/graduate/bioinformatics/), and some other university-related initiatives, such as the *Escola Gaúcha de Bioinformática* (https://www.ufrgs.br/egb/ ) and the course Programming for Bioinformatics with Python by the Federal University of Pará (UFPA) [[8]](https://paperpile.com/c/zTap62/LVmu). It is important to mention that initiatives exist to increase the critical mass in bioinformatics in Brazil and encourage students and future scientists to evolve in this field. They include the annual event X-meeting, organized by the Brazilian Association of Bioinformatics and Computational Biology (AB3C) (<https://ab3c.org.br/site/x-meeting> ) and the League of Brazilian Bioinformatics [[9](https://paperpile.com/c/zTap62/EMCD)]. In this scenario, we consider the Brazilian Python Workshop for Biological Data an original and important initiative to help teach future biologists to analyze their data and communicate with bioinformaticians, data analysts and biostatisticians.

# References

1. [Gauthier J, Vincent AT, Charette SJ, Derome N. A brief history of bioinformatics. Brief Bioinform [Internet]. 2019 Nov 27;20(6):1981–96. Available from:](http://paperpile.com/b/zTap62/3FeN) <http://dx.doi.org/10.1093/bib/bby063>

2. [DeMasi O, Paxton A, Koy K. Ad hoc efforts for advancing data science education. PLoS Comput Biol [Internet]. 2020 May;16(5):e1007695. Available from:](http://paperpile.com/b/zTap62/jLtU) <http://dx.doi.org/10.1371/journal.pcbi.1007695>

3. [Mulder NJ, Adebiyi E, Alami R, Benkahla A, Brandful J, Doumbia S, et al. H3ABioNet, a sustainable pan-African bioinformatics network for human heredity and health in Africa. Genome Res [Internet]. 2016 Feb;26(2):271–7. Available from:](http://paperpile.com/b/zTap62/Oi4W) <http://dx.doi.org/10.1101/gr.196295.115>

4. [De Las Rivas J, Bonavides-Martínez C, Campos-Laborie FJ. Bioinformatics in Latin America and SoIBio impact, a tale of spin-off and expansion around genomes and protein structures. Brief Bioinform [Internet]. 2019 Mar 22;20(2):390–7. Available from:](http://paperpile.com/b/zTap62/DkFY) <http://dx.doi.org/10.1093/bib/bbx064>

5. [Restrepo S, Pinzón A, Rodríguez-R LM, Sierra R, Grajales A, Bernal A, et al. Computational biology in Colombia. PLoS Comput Biol [Internet]. 2009 Oct;5(10):e1000535. Available from:](http://paperpile.com/b/zTap62/7hiD) <http://dx.doi.org/10.1371/journal.pcbi.1000535>

6. [Bassi S, González V, Parisi G. Computational biology in Argentina. PLoS Comput Biol [Internet]. 2007 Dec;3(12):e257. Available from:](http://paperpile.com/b/zTap62/qvnV) <http://dx.doi.org/10.1371/journal.pcbi.0030257>

7. [Neshich G. Computational biology in Brazil. PLoS Comput Biol [Internet]. 2007 Oct;3(10):1845–8. Available from:](http://paperpile.com/b/zTap62/2leC) <http://dx.doi.org/10.1371/journal.pcbi.0030185>

8. [de Araújo GS. From bioinformatics user to bioinformatics engineer: a report [Internet]. bioRxiv. 2020 [cited 2021 Apr 13]. p. 2020.08.03.225979. Available from:](http://paperpile.com/b/zTap62/LVmu) <https://www.biorxiv.org/content/10.1101/2020.08.03.225979v1.abstract>

9. [Carvalho LM, Coimbra N, Neves MRC, Fonseca N. League of Brazilian Bioinformatics: a competition framework to promote scientific training. bioRxiv [Internet]. 2020; Available from:](http://paperpile.com/b/zTap62/EMCD) <https://www.biorxiv.org/content/10.1101/2020.12.17.423357v1.abstract>
