# Supplementary material for: The experience of teaching introductory programming skills to bioscientists in Brazil
Source: PLoS Comput Biol. 2021 Nov 11;17(11):e1009534. doi: 10.1371/journal.pcbi.1009534 (PMC8584955; doi:10.1371/journal.pcbi.1009534)
Supplement: S2 Text — (DOC) [file pcbi.1009534.s008.doc]

Supplementary material 2

**Luíza Zuvanov^1,¶^**, **Ana Letycia Basso Garcia^2,¶^**, **Fernando Henrique Correr^2,¶^**, **Rodolfo Bizarria Júnior^3,9,¶^**, Ailton Pereira da Costa Filho**^4^**, Alisson Hayasi da Costa**^5^**, Andréa T. Thomaz**^6^**, Ana Lucia Mendes Pinheiro**^2^**, Diego Mauricio Riaño-Pachón**^7^**, Flavia Vischi Winck**^8^**, Franciele Grego Esteves^9^, Gabriel Rodrigues Alves Margarido**^2^**, Giovanna Maria Stanfoca Casagrande^10^, Henrique Cordeiro Frajacomo**^5^**, Leonardo Martins^11^, Mariana Feitosa Cavalheiro^12,13^, Nathalia Graf Grachet^14^, Raniere Gaia Costa da Silva^15^, Ricardo Cerri**^5^**, Rommel Thiago Juca Ramos^16^, Simone Daniela Sartorio de Medeiros^17^, Thayana Vieira Tavares^18^, **Renato Augusto Corrêa dos Santos^*,19,20^**

* [renatoacsantos@gmail.com](mailto:renatoacsantos@gmail.com)

**^¶^** these authors contributed equally

# A summary of the Brazilian Python Workshops for Biological Data

The Brazilian Python Workshop for Biological Data has been organized since 2017 as an initiative from volunteer-lead students to teach data science skills to students from life sciences. The core of the workshop is based on a combination of hands-on live coding sessions and guest lectures with the focus on data wrangling of real biological datasets using Python. All editions were hosted in different university campuses in Sao Paulo State, and were supported by professors at these hosting institutions (**S6 Table**), so that we could build a multi-disciplinary and diverse organizing committee each year, and also so we could reach students from different locations in the state.

The organizing team of the 2020 edition had 19 students and researchers from biological (93%) and exact sciences (7%) from seven institutions, most in São Paulo state (Brazil). In this edition, we were interested in documenting their achievements as being part of the organization of the event. For them, participating in the organization was important as a means to develop study groups (93.3%) and improve networking (73.3%). Additionally, they demonstrated an interest in expanding their own knowledge in Python programming, and becoming familiar with online communication platforms and open-source web applications for live coding.

Selection of enrolled students was based on the self-declaration of previous knowledge of programming languages, giving priority to those without any or with limited experience (**Fig 1A**). The goal of these selection criteria was to offer a hands-on experience to participants that were truly beginners in computer programming. We followed pre-established criteria to select participants from the undergraduate and graduate levels up to post-doc researchers and professors. The selection was based primarily on the participant’s letter of intention, which included a description of their research topic, and how their participation in the event would impact their research. We also asked participants to explain their expectations for the workshop, and those answers were also used as selection criteria. We gave priority to participants with interest in data analysis rather than in developing software tools, or developing advanced programming skills (**Fig 1B**). During the selection process we made a conscious effort to maintain equal gender participation.

The number of participants registered in the 2020 edition was the largest so far, and we attribute that to a better/larger broadcasting via social media and through partners such as the International Society for Computational Biology Regional Student Group (RSG-Brazil) (**S6 Table**). The virtual nature of this edition of the workshop due to the COVID-19 pandemic, plus the improved broadcasting, gave us the opportunity to reach a wider audience, with representatives of 12 states in Brazil (**Fig 2**). Hosting the event online attracted students that otherwise would not be able to participate. This perfectly aligned with our intent of reaching participants from as many different regions of Brazil as possible. These results provided us with perspectives that in a post-pandemic scenario the delivery of an online workshop would still be highly desired.

The schedule has changed over the editions (**S6 Table**). We moved from a two-day to a four-day workshop to provide a more comprehensive and in-depth knowledge in the basics of programming skills and applications in biological data. With these additional days, we invited speakers to demonstrate applications of Python programming in specific biological problems, which gave a more profound view of the applicability of programming to students (**S2 Table**). With more teaching time slots available in the agenda, we developed exercises and challenges to reinforce the knowledge acquired in the live coding sessions. This strategy also promoted interaction among participants. In 2017, students worked in groups to solve tasks and were rewarded with gifts. Similarly, in 2018, we implemented an individual problem-solving task with Python, in which a student resolving it in the shortest time was rewarded with a Python book. On the last day of the 2020 edition, we split students into groups to solve a common exercise, and they presented their work resolution at the end of the event.
