# Supplementary material for: The experience of teaching introductory programming skills to bioscientists in Brazil
Source: PLoS Comput Biol. 2021 Nov 11;17(11):e1009534. doi: 10.1371/journal.pcbi.1009534 (PMC8584955; doi:10.1371/journal.pcbi.1009534)
Supplement: S3 Text — (DOC) [file pcbi.1009534.s009.doc]

Supplementary material 3

**Luíza Zuvanov^1,¶^**, **Ana Letycia Basso Garcia^2,¶^**, **Fernando Henrique Correr^2,¶^**, **Rodolfo Bizarria Júnior^3,9,¶^**, Ailton Pereira da Costa Filho**^4^**, Alisson Hayasi da Costa**^5^**, Andréa T. Thomaz**^6^**, Ana Lucia Mendes Pinheiro**^2^**, Diego Mauricio Riaño-Pachón**^7^**, Flavia Vischi Winck**^8^**, Franciele Grego Esteves^9^, Gabriel Rodrigues Alves Margarido**^2^**, Giovanna Maria Stanfoca Casagrande^10^, Henrique Cordeiro Frajacomo**^5^**, Leonardo Martins^11^, Mariana Feitosa Cavalheiro^12,13^, Nathalia Graf Grachet^14^, Raniere Gaia Costa da Silva^15^, Ricardo Cerri**^5^**, Rommel Thiago Juca Ramos^16^, Simone Daniela Sartorio de Medeiros^17^, Thayana Vieira Tavares^18^, **Renato Augusto Corrêa dos Santos^*,19,20^**

* [renatoacsantos@gmail.com](mailto:renatoacsantos@gmail.com)

**^¶^** these authors contributed equally

[Final remarks about the virtual workshop during the COVID-19 pandemic and main takeaways](#_heading=h.1fob9te)

[Justifying initiatives like the Brazilian Python Workshop for Biological Data](#_heading=h.3znysh7)

[Transition from in-person to an online initiative](#_heading=h.2et92p0)

[Main takeaways from our personal and group experience](#_heading=h.tyjcwt)

[References](#_heading=h.3dy6vkm)

# Final remarks about the virtual workshop during the COVID-19 pandemic and main takeaways

## Justifying initiatives like the Brazilian Python Workshop for Biological Data

As a successful experience, we learned a lot from the Brazilian Python Workshop for Biological Data in terms of organization of such *ad hoc* initiatives and the educational aspects of teaching programming skills to bioscientists. Although the workshop has been successfully implemented, and the surveys showed promising perspectives, we must mention we are aware that debates exist on the real effect of short-term workshops, which is questionable whether any benefits in graduate research or socialization were directly associated with such initiatives [[1](https://paperpile.com/c/7sku0K/TsyH)]. [Garcia-Milian](http://paperpile.com/b/7sku0K/9gxs) et al. [[2](https://paperpile.com/c/7sku0K/9gxs)] verified that lack of training imposes difficulties to graduate and post-docs researchers in developing their projects. These authors also showed an attendee report arguing the efficiency of training depends on recent usage of the concepts following the event. [Feldon](http://paperpile.com/b/7sku0K/TsyH) et al. [[1](https://paperpile.com/c/7sku0K/TsyH)] also pointed out that learning progression is associated with previous skills required for specific training. To fulfill these expectations in our workshop, we selected participants to make sure their expectation was in alignment with the scope of the workshop, and they had the expected background (i.e., solid training in biology and basic ideas on what biological data are).

There is a second criticism regarding training workshops as a temporary solution for scientific programming, and that organizers should create long-term communities [[3](https://paperpile.com/c/7sku0K/hhO9)]. Our workshop provided a place that permitted the intellectual and personal growth of participants in cases where the students of one edition became an instructor or helper in a novel edition. In fact, a student in 2018, Luiza Zuvanov (an author in the current manuscript), participated in the organization of the event as a competent practitioner. In 2020, she helped to elaborate practical sessions with examples from her own research project, a practice we plan to maintain over the following years and editions.

## Transition from in-person to an online initiative

Our group was very enthusiastic about the workshop in 2020 and delivering programming skills to bioscientists, since we had great experiences and positive feedback in previous years (data not shown). However, the COVID-19 came as a high selective pressure for the organization since we had to shift from the in-person to the virtual mode this year. Also resulting from the pandemic, private industries and other partners we cultivated over the past years were not willing to sponsor the workshop in online format. On the other hand, we did not have the costs that are usually associated with local events like coffee-break, travel or accommodation expenses for organizers and speakers. Moreover, we used free versions of the available online tools before and during the workshop, and still as a consequence of the pandemic many companies released their online tools for testing or educational purposes. Additionally, having no costs allowed us to keep the philosophy of making the python workshop free to students, like in previous editions.

Another advantage of moving the event to a virtual format was the increased diversity in the group of students, organizers, and speakers. As an interdisciplinary field, data science and analysis of biological data requires knowledge of people with different training backgrounds. Therefore, although the workshop focuses on biological data, having organizers from computer science or engineering to help manage the data manipulation in the background (e.g., data collection via Google forms) is also strongly recommended. Moving to the online mode allowed us to invite speakers from different regions of the country or even Brazilians abroad, who promptly accepted our invitation. Interestingly, speakers were more prone to accept the invitation to join the team virtually, as it is easier to fit their schedules than an in-person workshop, as previously noticed [[4](https://paperpile.com/c/7sku0K/CoxO)]. Regarding the groups of selected participants, online mode helped us to reach different regions of our country. On the other hand, even though the online event enables more diversity among participants, we could not avoid the problem of reduced inclusion associated with requirements that participants have a computer with stable internet connection during the event [[5](https://paperpile.com/c/7sku0K/LqEi)]. Some of the participants and speakers reported internet connectivity issues from time to time, and hardware malfunctions (e.g., microphone issues).

Representation of participants with different backgrounds and socio-economic status was prioritized in the 2020 edition., We must stress that social aspects are very important in events like this that have high representation of students in general, as well as the concerns about ethics and integrity in science, that are unprecedented to improve future scientists, and must be incorporated at least subjectively. For example, equity, inclusion and diversity must be prioritized in the selection process . Maximizing the geographic locations of participants allowed us to provide inclusion, still ensuring the scientific excellence of participants and justified necessity of programming skills. In the last workshop, we achieved gender balance; however, for the next editions we recognize that a better assessment of the diversity of the participants, including ethnicity, sexual, economic and geographic background should be incorporated into the questionnaire to achieve a better representation of the Brazilian population.

Lastly, we should mention that we learned a lot about delivering programming skills online in this edition with what we have called “learn with learners”. We learned not only with the questions that were raised over the course of workshop development and with interactions between students and organizers. The daily feedback forms (surveys) also provided substantial material that allowed us to understand the evolution of the construction of the learning process itself. For instance, we identified difficulties associated with the use of concomitant digital platforms and the content of the material each day. Understanding that difficulties were more pronounced in the first two days (while students were still unfamiliar with platforms) helped us think about a better distribution and speed of information transmission and communication for the next workshop..

## Main takeaways from our personal and group experience

We can say for sure that personal interests, interactions, and empirically building the event over the years made great positive impacts in making this initiative successful, with “transferable skills” also playing an important role - also known as “portable skills,” are qualities that can be transferred from one job to another (e.g. organization or strong communication). For instance, the first edition of the workshop was experimental - we had very limited practical experience, but had substantial support from experienced faculties (Dr. Winck and Dr. Riaño-Pachón, co-authors in this manuscript). In addition, Renato Santos and Raniere Silva (both co-authors in this work) brought their experiences in the organization of short workshops that happened before 2017, mainly initiatives by the The Carpentry (https://carpentries.org/) community.

Another example of applications of “transferable skills” in our workshop is the strong research components, mainly due to the fact that all organizers are composed by students somehow involved in research in life sciences, and we were supported by experienced faculties also involved in research with biological data analysis. In 2020, we kept an active collection of literature and used Twitter to broadcast about the discoveries, improving the engagements of organizers and helping the team to bring innovations and real-time improvements to the workshop, visit “virtually” different experiences and raise concerns and discussion in the group. This culture of tweeting research was incorporated into the workshop by the previous experience of Renato’s internship abroad in Rokas Lab (Vanderbilt University, Nashville, US). Tweeting increased engagement of the organizers over time. For instance, 2020 was the first year implementing forms to collect information from participants (organizers and students) and discussions of the ELIXIR paper [[6](https://paperpile.com/c/7sku0K/xOkr)] that was selected to use in a tweet as a great example of how to conduct this research. During exercises and assignments by students, “transferable skills” were also very important during exchange of information and experience among themselves.

Transferable skills will also be very important to students in the long term. Interestingly, we identified in students’ responses not only evidences that they learned several aspects of programming skills (e.g. programming skills or data visualization) that they will be able to apply in a broader sense in their career, but they also reported more subjective information such as considering as valuable “didactics” (related to teaching and learning skills), “networking” (related to communication skills), or “things they could apply in diverse areas”. In addition, it was nice to notice that one student reported as valuable the “ability to think as a programmer”: one of the transferable skills that we expect that students assimilate is “computational thinking”, in particular because “all biology is computational biology” these days [[7](https://paperpile.com/c/7sku0K/XsqC)]. Reproducibility of research is extremely important in science, not only in experiments/wet-lab, but also in bioinformatics and data analysis in general. An example of initiative that improves reproducibility is the use of Jupyter notebooks [[8](https://paperpile.com/c/7sku0K/MbKk)]. We emphasized the importance of reproducible research during the workshop in 2020, listing some of the good practices during documentation of how data analysis was carried out with the notebooks. We highlight it as another example of “transferable skill” that students will be able to apply in their own research.

# References

1. [Feldon DF, Jeong S, Peugh J, Roksa J, Maahs-Fladung C, Shenoy A, et al. Null effects of boot camps and short-format training for PhD students in life sciences. Proc Natl Acad Sci U S A [Internet]. 2017 Sep 12;114(37):9854–8. Available from:](http://paperpile.com/b/7sku0K/TsyH) <http://dx.doi.org/10.1073/pnas.1705783114>

2. [Garcia-Milian R, Hersey D, Vukmirovic M, Duprilot F. Data challenges of biomedical researchers in the age of omics. PeerJ [Internet]. 2018 Sep 11;6:e5553. Available from:](http://paperpile.com/b/7sku0K/9gxs) <http://dx.doi.org/10.7717/peerj.5553>

3. [Stevens SLR, Kuzak M, Martinez C, Moser A, Bleeker P, Galland M. Building a local community of practice in scientific programming for life scientists. PLoS Biol [Internet]. 2018 Nov;16(11):e2005561. Available from:](http://paperpile.com/b/7sku0K/hhO9) <http://dx.doi.org/10.1371/journal.pbio.2005561>

4. [Salomon D, Feldman MF. The future of conferences, today: Are virtual conferences a viable supplement to “live” conferences? EMBO Rep [Internet]. 2020 Jul 3;21(7):e50883. Available from:](http://paperpile.com/b/7sku0K/CoxO) <http://dx.doi.org/10.15252/embr.202050883>

5. [Wright AM, Schwartz RS, Oaks JR, Newman CE, Flanagan SP. The why, when, and how of computing in biology classrooms. F1000Res [Internet]. 2019 Nov 5;8:1854. Available from:](http://paperpile.com/b/7sku0K/LqEi) <http://dx.doi.org/10.12688/f1000research.20873.2>

6. [Gurwitz KT, Singh Gaur P, Bellis LJ, Larcombe L, Alloza E, Balint BL, et al. A framework to assess the quality and impact of bioinformatics training across ELIXIR. PLoS Comput Biol [Internet]. 2020 Jul;16(7):e1007976. Available from:](http://paperpile.com/b/7sku0K/xOkr) <http://dx.doi.org/10.1371/journal.pcbi.1007976>

7. [Markowetz F. All biology is computational biology. PLoS Biol [Internet]. 2017 Mar;15(3):e2002050. Available from:](http://paperpile.com/b/7sku0K/XsqC) <http://dx.doi.org/10.1371/journal.pbio.2002050>

8. [Loizides F, Schmidt B. Positioning and Power in Academic Publishing: Players, Agents and Agendas: Proceedings of the 20th International Conference on Electronic Publishing [Internet]. IOS Press; 2016. 164 p. Available from:](http://paperpile.com/b/7sku0K/MbKk) <https://play.google.com/store/books/details?id=Lgy3DAAAQBAJ>
